# Supplementary material for: Content-rich biological network constructed by mining PubMed abstracts
Source: BMC Bioinformatics. 2004 Oct 8;5:147. doi: 10.1186/1471-2105-5-147 (PMC528731; doi:10.1186/1471-2105-5-147)
Supplement: Additional File 2 — The original results of the above study (non-essential files are deleted to keep the file size under the limit set by BMC bioinformatics). [file 1471-2105-5-147-S2.bz2 › chilibotAdditionalFile2/dip10/346ID10903799E4422/html/IL2RB_IL15.html]

 


 **IL2RB** and **IL15** 
  
Found 142 abstracts in PubMed, retrieved 10.  
 

 What does Google say? 
 PDF only 
| .edu only 

---

**Interactive relationship** (e.g. stimulation, inhibition, etc)

**Neutral relationship**- Interleukin IL 2 and IL 15  [ **IL15** ]  share the IL 2 receptor  [ **IL2RB** ]  betagamma c subunits IL 2Rbetagamma c but have specific, unique alpha receptor subunits.  Ref: 12453470 Cytokine, 2002
- IL 15  [ **IL15** ]  dependency is most extreme for the Ly49 subset of CD122  [ **IL2RB** ]  hi CD44 hi CD8 cells.  Ref: 12370255 J Exp Med, 2002
- The receptors for interleukin 2 IL 2 and interleukin 15  [ **IL15** ]  IL 15  [ **IL15** ]  in T cells share the IL 2R beta subunit CD122  [ **IL2RB** ]  and gamma C subunit but have private alpha subunits.  Ref: 12121224 Immunol Cell Biol, 2002
- In contrast to CD122  [ **IL2RB** ]  hi cells, the CD122  [ **IL2RB** ]  lo subset of CD44 hi CD8 cells is IL 15  [ **IL15** ]  independent.  Ref: 12370255 J Exp Med, 2002
- The major subset of CD122  [ **IL2RB** ]  hi CD44 hi CD8 cells is heavily dependent on IL 15  [ **IL15** ]  by three different parameters, namely 1 bystander proliferation induced via IFN induced stimulation of the innate immune system, 2 normal background proliferation, and 3 T cell survival.  Ref: 12370255 J Exp Med, 2002

**Non-interactive relationship** (e.g. studied together, co-existance, homology, etc.)

- likewise, being CD122  [ **IL2RB** ]  lo, CD44 hi CD4 cells are IL 15  [ **IL15** ]  independent.  Ref: 12370255 J Exp Med, 2002
- BACKGROUNDTo define whether immunosuppressive agents that block the interleukin IL 2 pathway could prevent activation induced cell death of activated T cells in the graft, we measured expression of IL 2, IL 2 receptor  [ **IL2RB** ]  alpha chain CD25, IL 15  [ **IL15** ] , Fas, and Fas ligand by real time reverse transcription polymerase chain reaction in cardiac allografts.METHODSWe characterized the phenotype of the infiltrating cells CD3, CD68, CD25 by immunohistochemistry.  Ref: 12777860 Transplantation, 2003
- Genes that were up regulated in cells exposed to D39 relative to those exposed to PLN included genes encoding proteins such as mannose binding lectin 1, lysozyme, alpha 1 catenin, cadherin 17, caspases 4 and 6, macrophage inflammatory protein 1beta MIP 1beta, interleukin 8 IL 8, monocyte chemotactic protein 3 MCP 3, IL 2 receptor  [ **IL2RB** ]  beta IL 2Rbeta, IL 15  [ **IL15** ]  receptor alpha IL 15Ralpha, interferon receptor 2, and prostaglandin E synthase.  Ref: 12654830 Infect Immun, 2003
